# Quality assurance of rifampicin-containing fixed-drug combinations in South Africa: dosing implications

**DOI:** 10.5588/ijtld.17.0697

**Published:** 2018-05-01

**Authors:** R. Court, M. T. Chirehwa, L. Wiesner, B. Wright, W. Smythe, N. Kramer, H. McIlleron

**Affiliations:** * Division of Clinical Pharmacology, Department of Medicine, University of Cape Town, Cape Town; † Clinical Research Centre, Faculty of Health Sciences, University of Cape Town, Cape Town, South Africa

**Keywords:** tuberculosis, dose, pharmacokinetic, bioequivalence, bioavailability

## Abstract

**SETTING:**

Rifampicin (RMP) drives treatment response in drug-susceptible tuberculosis. Low RMP concentrations increase the risk of poor outcomes, and drug quality needs to be excluded as a contributor to low RMP exposure.

**OBJECTIVES AND DESIGN:**

We performed an open-label, three-way cross-over study of three licensed RMP-containing formulations widely used in South Africa to evaluate the bioavailability of RMP in a two-drug fixed-dose combination tablet (2FDC) and a four-drug FDC (4FDC) against a single-drug reference. RMP dosed at 600 mg was administered 2 weeks apart in random sequence. Plasma RMP concentrations were measured pre-dose and 1, 2, 3, 4, 6, 8 and 12 h post-dose. The area under the concentration-time curve (AUC_0–12_) of the FDCs was compared to the single drug reference. Simulations were used to predict the impact of our findings.

**RESULTS:**

Twenty healthy volunteers (median age 22.8 years, body mass index 24.2 kg/m^2^) completed the study. The AUC_0–12_ of the 4FDC/reference (geometric mean ratio [GMR] 78%, 90%CI 69–89) indicated an average 20% reduction in RMP bioavailability in the 4FDC. The 2FDC/reference (GMR 104%, 90%CI 97–111) was bioequivalent. Simulations suggested dose adjustments to compensate for the poor bioavailability of RMP with the 4FDC, and revised weight-band doses to prevent systematic underdosing of low-weight patients.

**CONCLUSION:**

Post-marketing surveillance of in vivo bioavailability of RMP and improved weight band-based dosing are recommended.

## Abstract (FR)

**CONTEXTE:**

La rifampicine (RMP) conduit la réponse au traitement dans la tuberculose pharmacosensible. Des concentrations faibles de RMP augmentent le risque de résultats médiocres. Il faut exclure la qualité du médicament en tant que contribution à une exposition faible à la RMP.

**OBJECTIFS ET SCHÉMA:**

Nous avons réalisé une étude ouverte à trois voies et des groupes croisés de trois formulations homologuées contenant de la RMP et largement utilisées en Afrique du Sud, afin d'évaluer la biodisponibilité de la RMP dans des comprimés à doses fixes de deux médicaments combinés (2FDC) et dans des FDC de quatre médicaments (4FDC) vis-à-vis d'une référence contenant un seul médicament. Une dose de 600 mg de RMP a été administrée à 2 semaines d'intervalle en séquence aléatoire. Les concentrations plasmatiques de RMP ont été mesurées avant l'administration et à 1, 2, 3, 4, 6, 8 et 12 h après l'administration. Les zones sous la courbe de concentration dans le temps (AUC_0–12_) des FDC ont été comparées à la référence du médicament unique. Des simulations ont prédit l'impact des résultats.

**RÉSULTATS:**

Vingt volontaires en bonne santé (âge médian 22,8 ans ; indice de masse corporelle 24,2 kg/m^2^) ont achevé l'étude. L'AUC_0–12_ des 4FDC/référence (ratio de moyenne géométrique [GMR] 78%, IC90% 69–89) a mis en évidence une réduction moyenne de 20% de la biodisponibilité de la RMP pour les 4FDC. Les 2FDC et la référence (GMR 104%, IC90% 97–111) ont eu un résultat équivalent. Les simulations ont suggéré des ajustements de la dose pour compenser cette biodisponibilité médiocre de la RMP avec le 4FDC et ont révisé les posologies en fonction des zones de poids afin d'éviter un sous-dosage systématique des patients de poids faible.

**CONCLUSION:**

Il est recommandé d'exercer une surveillance après la mise sur le marché de la biodisponibilité in vivo de la RMP et d'améliorer la posologie basée sur les zones de poids.

## Abstract (ES)

**MARCO DE REFERENCIA:**

La rifampicina (RMP) es el factor fundamental de la respuesta al tratamiento en los casos de tuberculosis sensible a los medicamentos. Las bajas concentraciones de RMP aumentan el riesgo de desenlaces desfavorables. Es necesario excluir que la calidad de los medicamentos contribuya a bajas exposiciones a la RMP.

**OBJETIVOS Y MÉTODO:**

Se llevó a cabo un ensayo clínico sin enmascaramiento, con grupos cruzados y de tres vías, de tres formas farmacéuticas autorizadas que contienen RMP y cuya utilización está muy difunda en Suráfrica; su objetivo fue evaluar la biodisponibilidad de RMP en un comprimido con asociación en dosis fijas de dos principios (2FDC) y un FDC con cuatro principios activos (4FDC), en comparación con la referencia con un principio único. La RMP se administró en dosis de 600 mg con intervalos de 2 semanas en secuencias aleatorias. Se midieron las concentraciones plasmáticas de RMP antes de la dosis y 1, 2, 3, 4, 6, 8 y 12 h despúes de la misma. El área bajo la curva (de concentración plasmática y tiempo, AUC_0–12_) de las FDC se comparó con la referencia que contenía un principio activo único. Se modelizaron las repercusiones de los resultados.

**RESULTADOS:**

Completaron el estudio 20 voluntarios sanos (mediana de la edad 22,8 años y del índice de masa corporal 24,2 kg/m^2^). El cociente del AUC_0–12_ de 4FDC/referencia (cociente de la media geométrica [GMR] 78%; IC90% 69–89) indicó un promedio de 20% de disminución de la biodisponibilidad de RMP en el 4FDC. Se demostró la bioequivalencia del 2FDC y la referencia (GMR 104%; IC90% 97–111). Las simulaciones indicaron los ajustes de la dosis que permitirían compensar la escasa biodisponibilidad de RMP con la 4FDC y se revisaron las dosis con diferentes márgenes de peso, con el fin de evitar la infradosificación sistemática de los pacientes con bajo peso.

**CONCLUSIÓN:**

Se recomienda que se practique la vigilancia posterior a la comercialización de la biodisponibilidad de RMP in vivo y que se mejore la dosificación en función de los intervalos de peso.

RIFAMPICIN (RMP) is a key drug driving treatment response in drug-susceptible tuberculosis (TB). Low RMP exposures are associated with poor outcomes, including the development of drug resistance.^1^ Plasma RMP concentrations have been shown to vary between and within patients.^2^ Reduced plasma concentrations of RMP have also been associated with the presence of polymorphisms in the *SLCO1B1* gene, which encodes organic anion-transporting polypeptide 1B1,^3^,^4^ and with clinical factors such as human immunodeficiency virus (HIV) infection,^5^ low body mass index (BMI)^6^ and male sex.^6^ Formulation is another determinant of drug exposure,^7–9^ although the extent to which the quality of drug formulations affects pharmacokinetic (PK) variability has not been well explored. The bioavailability of RMP in fixed-drug combination (FDC) tablets is particularly problematic.^10–13^ Causes of altered bioavailability of RMP in FDCs may include changes in the crystalline structure during the manufacturing process, adsorption by excipients and particle size.^11^,^14^,^15^ Packaging and storage conditions are also important to prevent ex vivo decomposition of RMP in the FDC.^16^

There are no validated correlates of bioavailability in humans.^12^,^13^ However, after licensing, independent assurance of product quality relies on good manufacturing practice inspections, for example as part of the World Health Organization (WHO) prequalification programme. The proposed requirement for comparative bioavailability studies of RMP on the submission of tenders^17^ does not appear to be widely applied. Following their endorsement of the replacement of single-drug products with FDCs for the first-line treatment of TB, the WHO and the International Union Against Tuberculosis and Lung Disease recommended continuous surveillance of FDC quality.^17^ However, in the absence of validated simpler and cheaper methods than bioequivalence testing in humans, such efforts have been limited. It has also been unclear who should be responsible for implementing and funding post-marketing surveillance.

TB treatment success rates for South Africa remain below the global average. In response to demands that drug quality be excluded as a contributing factor, we performed a bioequivalence study on two FDCs registered for more than 15 years that are widely used to treat drug-susceptible TB in South Africa.

## STUDY POPULATION AND METHODS

We performed a single dose, open-label, three-way cross-over bioavailability study in healthy volunteers, with a 2-week wash-out period between each 600 mg dose of RMP. The two-drug FDC (2FDC) containing RMP/isoniazid (INH) 150/75 mg, Rimactazid^®^ (Sandoz, Kempton Park, South Africa), and the four-drug FDC (4FDC) containing RMP, INH, pyrazinamide (PZA) and ethambutol (EMB) 150/75/400/275 mg, Rifafour e-275^®^ (Sanofi-Aventis, Johannesburg, South Africa), were compared with a single-drug reference product, Rimactane^®^ (Sandoz). Eligibility criteria included healthy adult patients between the ages of 18 and 55 years with a BMI of 19–30 kg/m^2^. We excluded volunteers with a history of TB, as well as pregnant or breastfeeding women. Use of medications potentially interacting with the study drug were prohibited in the month before recruitment, to exclude the possibility of a drug-drug interaction. Treatment sequence was randomised.

Eligible participants were admitted to the research ward overnight before the respective study doses. After a standard evening meal, the participants fasted overnight until 2 h post-dose. Water was allowed ad lib except within 1 h before and after the RMP dose. Dosing was strictly observed, and the drugs were taken with 250 ml of water. Blood was sampled for RMP plasma concentrations pre-dose and at 1, 2, 3, 4, 6, 8 and 12 h post-dose. The blood samples were immediately centrifuged, and aliquoted plasma was stored at −70°C within 60 min of sampling.

Safety monitoring included a clinical assessment and measurement of alanine aminotransferase 24 h before each study treatment dose. Participants were also encouraged to report any adverse effects during the study period. A full blood count and liver and renal function tests were repeated within 2–7 days after the last study treatment dose. All adverse events were graded according to the classification published by the Division of AIDS of the National Institutes of Health, Bethesda, MD, USA.^18^

RMP plasma concentrations were determined using a validated liquid chromatography tandem mass spectrometry method developed at the Division of Clinical Pharmacology, University of Cape Town (UCT), Cape Town, South Africa, as previously described.^9^ The assay was validated over the concentration range of 0.117–30 μg/ml. The combined accuracy and precision statistics of the limit of quantification, low-, medium- and high-quality controls (three validation batches, no 18) were between 101% and 107%, and 2.7% and 3.7%, respectively.

### Pharmacokinetic and statistical analysis

Considering that participants were healthy volunteers and that there was a 2-week washout period between doses, we treated plasma RMP concentrations below the lower level of quantification as zero. We performed non-compartmental analysis using Stata v 13.1 (StataCorp, College Station, TX, USA) to compute peak concentration (C_max_), area under the concentration-time curve to 12 h (AUC_0–12_) and area under the concentration-time curve to infinity (AUC_∞_) for each concentration-time curve. The trapezoidal rule was applied to compute AUC, and the exponential extrapolation option was selected to derive AUC_∞_. The log-transformed C_max_, AUC_0–12_ and AUC_∞_ for the two FDCs were compared with the RMP-only reference product in paired *t*-tests. The geometric mean ratio (GMR) point estimates and 90% confidence intervals (CIs) were calculated for RMP's log-transformed C_max_, AUC_0–12_ and AUC_∞_ for the 4FDC/reference and the 2FDC/reference ratios, respectively, using published bioequivalence criteria.^19^ As bioequivalence is assessed using log-transformed PK measures in the recommended range of 80–120% with 90%CIs, this is equivalent to performing two one-sided tests of hypotheses at a 5% level of significance (i.e., 95%CI). Analysis of variance (ANOVA) was used to account for period or sequence effects.

### Simulations

Using a published population PK model describing RMP PK in South African TB patients assumed to be treated with compliant formulations,^20^ we used simulation to predict RMP exposure in patients by WHO-recommended dosing weight bands. Based on the findings of this study, we simulated exposures with and without a 20% reduction in bioavailability. The simulations were based on the weight, height and sex distributions among 1092 African patients with drug-susceptible TB in our database.

### Ethics

The study (clinicaltrials.gov NCT02953847) was approved by the Human Research Ethics Committee, Health Sciences Faculty, University of Cape Town, Cape Town, South Africa (HREC REF: 272/2015), and the Medicines Control Council, Pretoria, South Africa (REF: N2/19/8/2). Informed consent was provided by each participant in the language of their choice after screening. A translator was provided for the informed consent process if necessary.

## RESULTS

We screened 40 volunteers and recruited 28 participants. Volunteers who failed screening for medical reasons were referred for appropriate care. Five volunteers withdrew consent for personal reasons. We thus randomised 23 participants, who received at least one dose of the study treatment. One participant with a grade 1 hypersensitivity reaction was withdrawn after the first dosing occasion, and an additional two participants withdrew for personal reasons before study completion. The characteristics of the 20 participants who completed the study are shown in Table 1. No grade 2 or higher adverse events were observed.

**Table 1:**
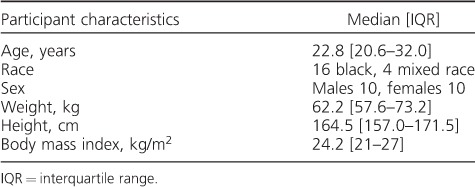
Participant characteristics of 20 healthy participants in a cross-over study of three formulations containing 600 mg of rifampicin

### Pharmacokinetics

The PK measures of the three formulations of RMP are given in Table 2. The comparative bioavailability of the two-drug and four-drug FDCs to the single-drug reference product are shown in Table 3. While the 90%CIs of the 2FDC/reference product ratio fell within the 80–125% range, the bioavailability of the 4FDC was approximately 20% lower than that of the reference. ANOVA on the log-transformed PK measures did not find any statistically significant period or sequence effects. We excluded two PK profiles: one for the reference product, which was determined to be implausible (incorrect labelling of the sample tubes was likely), and one for the 2FDC, as the final data point was elevated, resulting in a spuriously high half-life and AUC_∞_.

**Table 2:**
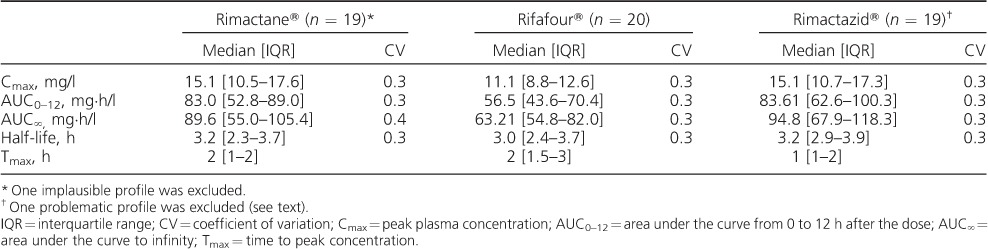
Pharmacokinetic measures of interest by study treatment in the 20 participants completing a cross-over study of three formulations containing 600 mg of rifampicin

**Table 3:**
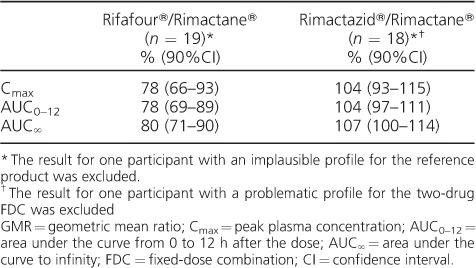
GMRs of C_max_, AUC_0–12_ and AUC_∞_ for the FDC/reference product

### Simulations

**Figure 1.**
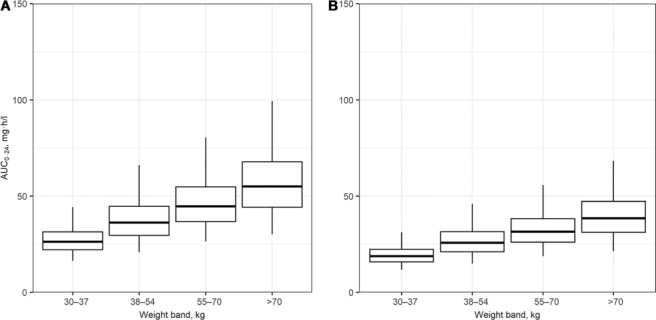
Distributions of the predicted AUC_0–24_ based on simulations in African patients with drug-susceptible tuberculosis dosed according to WHO-recommended weight-band guidelines. Panel A = patients are assumed to have been dosed with fully compliant formulations. Panel B=a 20% reduction in the bioavailability of RMP is included in the simulations. Weight bands: 30–37 kg, 2 tablets; 38–54 kg, 3 tablets; 55–70 kg, 4 tablets; >70 kg, 5 tablets. Median = box midline; interquartile range = upper and lower bounds of boxes; 95% range = upper and lower bounds of whiskers. AUC_0–24_ = area under the RMP concentration-time curve to 24 h; RMP = rifampicin.

**Figure 2.**
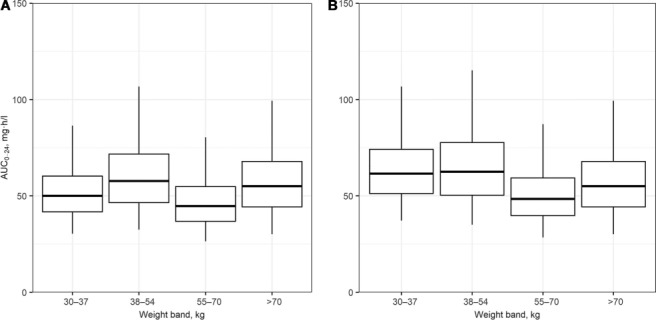
Simulated steady-state AUC_0–24_, with proposed adjusted weight-band dosing to compensate for low RMP bioavailability in patients with weight < 55 kg. Panel A = dosed as a fully compliant four-drug fixed-dose combination; Panel B = assuming a 20% decrease in bioavailability across all weight bands, with our proposed adjusted weight-band dosing plus an additional single 150 mg dose of RMP with 100% bioavailability dosed in all weight bands. Weight bands: 30–37 kg, 3 tablets; 38–54 kg, 4 tablets; 55–70 kg, 4 tablets; >70 kg, 5 tablets. Median = box midline; interquartile range = upper and lower bounds of boxes; 95% range = upper and lower bounds of whiskers. AUC_0–24_ = area under the RMP concentration-time curve to 24 h; RMP = rifampicin.

Figure 1, Panel A illustrates the steady-state AUC_0–24_ of RMP per WHO-recommended 4FDC weight-band dosing guidelines in South Africa; a simulated 20% decrease in bioavailability per weight band is shown in Figure 1, Panel B. Our proposed adjustment (adding one FDC tablet) to the weight-band dosing guidelines to compensate for the decrease in RMP bioavailability in patients with low weight (30–54 kg) using fully compliant formulations is shown in Figure 2, Panel A. Figure 2, Panel B shows the steady-state AUC_0–24_ of RMP dosed as Rifafour with our proposed adjusted weigh-band dosing, including an additional single 150 mg dose of RMP dosed in all weight bands to compensate for a simulated 20% decrease across all weight bands.

## DISCUSSION

We report a reduction of approximately 20% in RMP bioavailability in the 4FDC, Rifafour, compared to the single-drug reference product, Rimactane. However, the bioavailability of the 2FDC, Rimactazid, was comparable with the reference product. Approximately 450 000 new cases of TB occur in South Africa each year, the majority of whom have drug-susceptible TB^21^ and will therefore be treated with the 4FDC Rifafour during the intensive phase of treatment. Rifafour currently constitutes 100% of the South African Department of Health (DOH) procurement as the RMP-containing 4FDC, and was therefore selected as the study 4FDC. We selected Rimactazid as the 2FDC (approximately 30% of the DOH procurement for the continuation phase of drug-susceptible TB) because it is manufactured by a different drug company.

This report adds to the growing body of evidence highlighting the reduced bioavailability of RMP in FDCs described in multiple settings, including recently used formulations,^10–13^,^22^ and highlights the need for regular bioequivalence testing of RMP-containing FDCs. There are several possible reasons for the variable exposure of RMP. Polymorphisms in the crystalline formulations of RMP occurring as a result of the processing and tableting of RMP during manufacture are thought to result in varying plasma concentrations.^23^ RMP also degrades in an acidic environment when INH is present, although the degradation varies significantly between FDCs, suggesting the influence of formulation factors and storage conditions.^23^,^24^ Adsorption by excipients and particle size,^11^,^14^,^15^ as well as inappropriate packaging, are other factors that may affect the decomposition and absorption of RMP in the FDC.^16^

As low RMP plasma concentrations have been associated with poor clinical outcomes,^1^,^21^,^22^ it is critical that the quality of RMP-containing formulations is maintained. While the efforts of the WHO and national regulatory authorities have undoubtedly improved the quality of TB drug products approved for use in treatment programmes, it is clear that formulation and batch effects persist.^10^,^12^,^22^,^25^ While ongoing quality surveillance is advocated, these efforts are limited, especially for RMP.^26–28^

An appropriate response to our finding of reduced RMP bioavailability in the 4FDC is not straightforward. It is unclear whether the finding applies to multiple batches; we tested a single batch. We have reported our findings to the national medicines regulatory authority, which is working with the manufacturers to resolve the issue. Replacement of the formulation in the programme would be challenging, given the size of the procurement. On the other hand, it is not impossible that the reference formulation and the 2FDC, which are both from the same manufacturer, have above requisite bioavailability for RMP. However, we feel this is unlikely given the predisposition of FDCs, and the more complex FDCs in particular, to bioavailability problems.

To facilitate procurement, the WHO recommends weight-band dosing with 2–5 FDCs (RMP, INH, EMB and PZA) during the intensive phase of treatment for drug-susceptible TB.^29^ As clearance per unit of weight increases as body size decreases,^30^ when a standard dose/kg of body weight is prescribed, patients in the lower weight bands achieve lower drug exposures.^6^,^31^ For RMP, clearance is more closely correlated with fat-free mass than total body weight. The reported associations of HIV infection and male sex with reduced RMP exposures may be confounded by the effects of wasting or a relatively high fat-free mass, respectively. To compensate for reduced exposures in the lower weight bands, the number of FDCs should be increased to provide more uniform exposures and simplified weight bands (Figure 2, Panel A). Using population PK models, we confirmed that the adjustment is also appropriate for INH, PZA and EMB.^32^ In addition, using simulations, we found that adding a 150 mg RMP capsule or tablet across all weight bands would reasonably overcome the effect of a 20% reduction in bioavailability. Given that doses of up to 20 mg/kg^33^,^34^ and higher^35^ have been well tolerated, the risks associated with underdosing RMP in this context are likely to outweigh the risks of increasing the doses to compensate for the difference in bioavailability, should the 20% reduction in bioavailability be limited to some batches.

Our study had several limitations. First, simulations were based on RMP bioavailability at steady state, which includes allowance for auto-induction and nonlinearity of the dose-exposure relationship.^20^ As this could result in a smaller difference between the AUCs of the respective products, it is possible that the simulations were based on a slightly overestimated reduction in bioavailability once auto-induction was complete. Second, our study was not designed to investigate the clinical outcomes of low RMP concentrations. Given the effect of low RMP exposure on clinical outcomes, we regard a 20% reduction in RMP bioavailability as being significant. However, we are unable to quantify the effect that a 20% reduction would have on treatment response in drug-susceptible TB. Third, we have assumed that the additional 150 mg of RMP recommended to compensate for reduced bioavailability in the RMP-containing FDC (Figure 2) has a relative bioavailability of 100% compared to a reputable product accepted by the regulatory authority as being suitably bioequivalent.^17^

## CONCLUSION

As RMP is a key drug driving treatment response in TB, and in light of the risk of emerging drug resistance with suboptimal drug exposure, it is important that the quality of RMP-containing formulations, especially FDCs, be closely monitored. Comparative bioavailability studies in healthy normal volunteers are the current standard for assuring the quality of RMP-containing products. Simpler, cheaper methods should be explored and validated. Models can be used to estimate PK implications in patients resulting from changes in bioavailability and to predict compensatory dose adjustments. We recommend that the current treatment guidelines be adjusted to increase the drug doses in the lower weight bands, and that supplemental RMP be used together with Rifafour in the South African National Tuberculosis Control Programme pending replacement of the 4FDC with one of demonstrated bioequivalence.
